# Effects of Non-Ablative Solid-State Vaginal Laser (SSVL) for the Treatment of Vulvovaginal Atrophy in Breast Cancer Survivors after Adjuvant Aromatase Inhibitor Therapy: Preliminary Results

**DOI:** 10.3390/jcm12175669

**Published:** 2023-08-31

**Authors:** Daniel M. Lubián-López, Carmen A. Butrón-Hinojo, Salomón Menjón-Beltrán, Ernesto González-Mesa, Silvia Tapiador-Albertos, Bibiana Rodríguez-Jiménez, Gabriel Fiol-Ruiz

**Affiliations:** 1 Department of Obstetrics and Gynecology, University Hospital of Jerez de la Frontera, 11407 Cádiz, Spain; aisha.butronhinojo@gmail.com; 2 Department of Obstetrics and Gynecology, Viamed Bahía de Cádiz Hospital, 11130 Cádiz, Spain; 3 Department of Obstetrics and Gynecology, Faculty of Medicine, University of Cádiz, 11003 Cádiz, Spain; 4 Department of Obstetrics and Gynecology, Regional University Hospital of Granada and Inagor Women’s Clinic, 18001 Granada, Spain; smenjon@gmail.com; 5 Department of Obstetrics and Gynecology, Regional University Hospital of Málaga, 29001 Málaga, Spain; egonzalezmesa@gmail.com; 6 Department of Obstetrics and Gynecology, Alboran CMM Women’s Clinic, 04001 Almería, Spain; silviatapiador@clinicaalboran.es (S.T.-A.); bibianarodriguez@clinicaalboran.es (B.R.-J.); gabrielfiol@clinicaalboran.es (G.F.-R.)

**Keywords:** non-ablativesolid-state vaginal laser (SSVL), vulvo-vaginal atrophy (VVA), breast cancer survivors, aromatase inhibitors

## Abstract

Background: One of the side effects of anti-estrogen treatments in breast cancer survivors (BCSs), especially with aromatase inhibitor (AI) treatment, is the frequent appearance of vulvo-vaginal atrophy (VVA). We aim to evaluate the efficacy, safety and feasibility of a new type of non-ablative Solid-State Vaginal Laser (SSVL) treatment in BCSs with VVA. Methods: A total of 30 BCSs with a history of AI use and symptoms of VVA were treated with a non-ablative SSVL (LASEmaR 1500™-EUFOTON)in this non-randomized pilot study. The effects of the laser have been evaluated at baseline, 10 wk and 24 wk using a visual analogue scale (VAS), the Vaginal Health Index (VHI), the Vulvar Health Index (VuHI), the Female Sexual Function Index (FSFI), the EORTC QLQ-BR23, the Vaginal Maturation Index (VMI) and vaginal pH. Results: At 10-week follow-up vs. baseline there were no statistically significant differences in FSFI, lubrication and EORTC QLQ-BR23. In all the subjective (dyspareunia, VHI, VuHI, FSFI, QLQ) and objective parameters (VMI and pH) there was a statistically significant improvement at the 6-month follow-up. Satisfaction was very high (4.7 out of 5), with 95.7% of patients being satisfied, more than or very satisfied. Conclusions: Preliminary results of SSVL treatment of VVA and dyspareunia in BCSs after AI treatment suggest clinical improvement, without relevant side effects and with a high degree of satisfaction

## 1. Introduction

Vulvovaginal atrophy (VVA) is the main component of genitourinary syndrome of menopause(GMS) [1]. It is often associated with other vulvovaginal symptoms such asvaginal dryness and dyspareunia and, less frequently, recurrent urinary infections [2]. All these conditions, as it is well known, negatively affect the quality of life (QoL) of many women affected by this syndrome [3]. It is the estrogen loss either in postmenopausal women or inyoung patients with bilateral ovariectomy, as well as inpremenopausal women treatedfor breast cancer with chemotherapy or with antiestrogens, the so-called breast cancer survivors(BCSs) [4], that results in VVA.

Although GMS affects more than half of postmenopausal women, it is more prevalent in BCSs [5,6], and few are the women diagnosed and treated [7,8]. Postmenopausal BCSs treated with aromatase inhibitors (AIs) tend to have a more severe form of VVA than tamoxifene(TAM) users [9], with higher rates of vaginal dryness and dyspareunia [3,10,11]. According to our experience [12], we observed a high prevalence of sexualinactivity among BCSs regardless of AI use, but AI users had a significantly higher prevalence of female sexual dysfunction, worse QoL and higher anxiety.

Ideally, the optimal therapy for estrogen-deficiency symptoms is systemic or topical estrogen administration [13]. However, oral estrogens are contraindicated [14] and vaginal estrogens are not generally advised on BCSs [15]. The safety of other drugs such asoral ospemiphenehas not been established yet.

Because of its promising preliminary results, vaginal laser therapy (Carbon Dioxide (CO_2_) laser or Erbium-doped Yttrium Aluminum Garnet—Er:YAG la—) is being used for VVA in BCSs, but efficacy and safety data from clinical trials are lacking [16]. In 2018, FDA issued a safety communication warning patients about the risks associated with the use of these ablative devices, including vaginal burns, scarring, pain during sexual intercourse and recurring/chronic pain [17]. In 2020, the American College of Obstetricians and Gynecologists [18] and the American Urogynecologic Society [19] concluded that additional large clinical trials are needed to determine the benefits, risksand cost-effectiveness of ablative laser therapy for VVA in healthy women and in BCSs.

The aim of this study was to evaluate the efficacy and safety of a new type of non-ablative laser treatment (Solid-State Vaginal Laser—SSVL) on the vaginal epithelium, as well as on dyspareunia, sexuality and quality of life of BCSs women treated with AIs as adjuvant therapy and affected by VVA and dyspareunia.

## 2. Materials and Methods

### 2.1. Study Design 

A multicenter, single-arm, non-randomized pilot study was conducted in menopausal women with estrogen-dependent breast cancer who had completed adjuvant therapy with aromatase inhibitors at least six months before inclusion in the study, aged 45 to 65 years, and who presented symptoms of VVA (vaginal dryness, irritation, pain, etc.) and dyspareunia not susceptible to systemic or local estrogen treatment. In addition, participants had to meet the following inclusion criteria: breast cancer stage I, II, estrogen receptor-positive, disease-free survival, more than 12 months of amenorrhea and FSH > 40 mUI/mL, coital sexual activity with the same partner in the last 6 months, dyspareunia (pain during intercourse) and ability to understand the study and sign the informed consent.The following exclusion criteria are considered: use of systemic (MHT) or localhormonal treatment (LHT) in the six months prior to inclusion in the study, use of vaginal moisturizers in the 30 days prior to the start of treatment, use of vaginal lubricants for sexual intercourse in the 10 days prior to the start of treatment, acute or recurrent urinary tract infections, uterine prolapse, cystocele or rectocele higher than grade II, previous vulvovaginal surgery or radiotherapy (RT), collagenosis or autoimmune disease, serious or chronic disease (regardless of breast cancer) that may interfere with compliance with the study orpsychiatric illness or mental disorder that makes follow-up difficult or that conditions the signing of the informed consent.This study was conducted according to the guidelines of the Helsinki Declaration (https://www.wma.net/what-we-do/medical-ethics/declaration-of-helsinki/ (accessed on 23 November 2021), revised in 2013, and resolution 196/96 of the National Health Council on Research Involving Human Subjects. Approval fromthe University Hospital of Torrecardenas ethics committee was obtained. Study code: EETL-SSVL-12/2020. After a detailed explanation of the study protocol, all the participants signed the written informed consent.

### 2.2. Study Protocol

**Figure 1:**
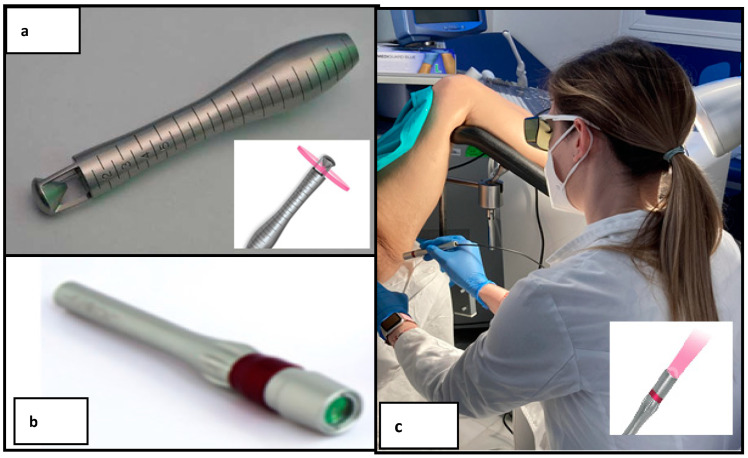
(**a**) Vaginal probe (SSVL handpiece: LADYLIFT). (**b**) External biostimulation head. (**c**) Dr. Butrón (gynaecologist) performing a vulvar introitus treatment (introitus, clitoris, fork, labia minora and labia majora) with the SSVL laser.

All patients have been treated with a non-ablative SSVL (LASEmaR 1500™-EUFOTON) with a wavelength of 1470 nm using a fluence (laser energy emitted per unit area) of 10–15 J/cm^2^. It is a medical device approved by a CE0476-certified body and under directives 93/42/CEE and 2007/47/CE. The SSVL handpiece (LADYLIFT), used to perform the treatment, is an internal vaginal probe developed for a gentle introduction with continuous radial light emission working at 360° in the vaginal canal (Figure 1a).Its spot size is larger than the spot size of other vaginal lasers (CO_2_, Erb-Yag) and this feature allows a greater depth of penetration improving energy absorption on the target. In particular, this laser uses a laser wavelength with an adequate mixture of absorption by water and penetration of tissue without generating ablation. In the vulva, an external biostimulation head has been used to generate collagen and vascularization in the external zone and introitus of the patient, globally improving the internal and external parts of the patients who have participated in this study (Figure 1b,c). Selective photothermolysisis achieved because this laser uses a wavelength with a proper mix between water absorption and tissue penetration without creating ablation. This makes it capable of penetrating 2–3 mm deep, without the need to burn the external tissue. Thanks to its penetration, it allows working at very low powers and energies for tissue regeneration, respecting the vaginal mucosa and reaching the lamina propria where it raises the temperature to 45 °C for the formation of new collagen. 

According to our protocol, in this study, each treatment session included a laser application every 15 to 20 days for 4 sessions in about 2months. A power of 2–3 watts wasused with energies of 10–15 joules for 5 min in the vagina and 5 min in the vulva. Time points of the study were at baseline (T1), at week 2 (T2), at week 4 (T3), at week 6 (T4), at week 10, a follow-up after 4 weeks from the last laser application (T5) and a final follow-up at 24 weeks (T6). Laser applications were performed at T1, T2, T3 and T4.The treatments were performed on an outpatient basis without the need for special preparation of the patient and without any type of anesthesia and with immediate return to daily life. The SSVL machine is very easy to use and transport due to its small size. After each treatment the patients were advised not to have sexual activity and not to wear tight clothing and/or play sports that would rub the treated area for 1 week. 

### 2.3. Assessment Tools

A standardized gynecologic clinical assessment for the presence of VVA was performed by 3 experienced clinical investigators in vulvovaginal pathology (1 per center) consisting of a physical examination, with additional tests (including the pelvic examination, vaginal cytology and a vaginal pH assessment) carried out in accordance with routine gynecologic clinical practice. The investigators filled out all signs of VVA to calculate the Vaginal Health Index [20], and the Vulva Health Index [21]. Objective criteria for changes in the vaginal mucosa have been determined by means of vaginal cytology and vaginal pH measurement, used as a part of the VHI calculation, at basal (T1), at 10 weeks (T5) and at 24 weeks (T6).The Vaginal Maturation Index (VMI), which shows increased ratios of intermediate and parabasal cells compared to superficial cells, was established by cytological examination using light microscopy. Gynecological cytologists were blinded to the randomization group and sample sequence (before or after treatment). VMI scores range from 5 to 25, with higher scores indicating better vaginal health status [22]. To assess vaginal pH, a piece of litmus paper was placed on the lateral vaginal wall until moistened. A pH of 4.6 or greater indicates vulvovaginal atrophy (VVA), assuming the patient does not have bacterial vaginosis. Premenopausal women without VVA typically have a pH of 4.5 or less [23]. All subjective clinical parameters have been evaluated using different tools at baseline (T1), after the final treatment (T5) and at 24 weeks (T6).The Visual Analog Scale (VAS) was performed for assessment of the severity of the dyspareunia.The scale’s left extremity indicates the complete absence of symptoms (0) and the right extremity indicates the worst possible symptom (10), and women rated the symptoms from 0 to 10. The Vaginal Health Index (VHI), a quantitative assessment of vaginal health, was performed by the investigator to assess changes in vaginal elasticity, vaginal secretions, epithelial mucous membrane integrity and vaginal humidity, along with pH as the only objective criteria. Each parameter was graded from 1 to 5 with a total score ranging from 5 to 25, with lower scores corresponding to greater urogenital atrophy, a total score ≤ 15 being atrophic [20]. The Vulva Health Index (VuHI) evaluates labia, urethra, clitoris, introitus as well as elasticity and pain during intercourse; the total score ranges from 0 to 24, with higher scores corresponding with greater vulvar atrophy. If the Vulva Health Index is over 8 or there is score of 3 (severe) in any category, vulvar atrophy is suggested [21]. The Female Sexual Function Index (FSFI) is a 19-item, multidimensional self-report instrument for assessing the key dimensions of sexual function in women. It investigates six different domains of sexual function: satisfaction, desire, lubrication, pain, arousal and orgasm. FSFI total score is scaled from 2 to 36, 2 being the worst sexual function. A cut off of 26.5 points or lower identifies women at risk of female sexual dysfunction [24]. It has been validated for BCSs [25]. A widely validated breast cancer-specific quality of life (Qol) questionnaire (EORTC QLQ-BR23) was used [26]. The raw scale and single-item scores on the QLQ-BR23 are linearly converted to a 0–100 scale. For the functional scales and single items (body image, sexuality and future perspective), higher scores reflect improved function. After every laser session, immediate adverse effects (AEs) such as vaginal bleeding, were evaluated. Vaginal bleeding during laser application is considered any bleeding from the vaginal walls and/or cervix secondary to atrophy (not vaginitis) and having ruled out an endometrial origin. Tolerance (vulvar or vaginal pain) to treatment was assessed using the Visual Analog Scale (0–10) at T1 and T4. Vaginitis or vaginal/vulvar burns were assessed as existing or non-existing at T1, T5 and T6. The level of satisfaction was assessed using a Likert scale, with scores ranging from “Not at all Satisfied”, “Partly Satisfied”, “Satisfied”, “More than Satisfied”, “Very Satisfied”, numbering 1 to 5 as an interval scale.

### 2.4. Statistical Analysis

Statistical analysis was performed with IBM SPSS Statistics version 28.0.1 (2022). The normal distribution of the sample was evaluated using the Shapiro–Wilk test. Analyses of the subjective and objective outcomes were performed. Continuous variables were compared using the paired-samples *t* test and presented as mean and standard deviation (SD). Contingency tables were assessed using the Fisher exact test. A two-sided *p*  < 0.05 was considered statistically significant.

## 3. Results

A total of 38 women with estrogen-dependent breast cancer and a history of IA use who presented symptoms of VVA and dyspareunia were informed about the study. Two (5.2%) refused to participate (one for fear of laser treatment and another because she was ill with COVID-19).Among the 36 patients who agreed to participate and were evaluated for eligibility, 30 (83.3%) met the criteria to participate in the study. Of these, 27 completed the 10-week follow-up and were included in the study, and 24 patients completed the 6-month follow-up. The mean [SD] age was 57.17 (3.7) years. The demographic characteristics of the patients are shown in Table 1.

Table 2 shows the efficacy outcomes before and after laser treatment at 10-week and at 24-week follow-up. There were significant improvement at 10-week follow-up in different subjective outcomes as dyspareunia from a mean (SD) of 7.5 (1.6) points at baseline to 3.4 (2.4) points at the 10-week follow-up (*p* < 0.001); Vaginal Health Index (VHI) from 10.6 (3.3.) to 17.1 (3.9), *p* < 0.001; Vulvar Health Index (VuHI) from 9.7 (3.1) to 3.9 (3.2), *p* < 0.001 and in all of the objective outcomes, so vaginal pH improved from a mean (SD) of 6.5 (0.8) points at baseline to 6.3 (1.0) at the 10-week follow-up (*p* < 0.001) and Vaginal Maturation Index (VMI) from 5.7 (9.2) to 17.2 (16.7), *p* < 0.001. There were no statistically significant differences between baseline and 10-week follow-up in EORTC QLQ-BR23 from a mean (SD) of 39.1 (8.4) to 37.8 (6.4), *p* = 0.29; FSFI from 18.8 (8.1) to 21.05 (4.2), *p* = 0.13; and lubrication from 2.7 (1.4) to 3.1 (1.5), *p* = 0.19.

In all the subjective and objective parameters there were a statistically significant improvement at the 6-month follow-up, being greater than at 10-week from the start of treatment (Table 2). Dyspareunia improved from a mean (SD) of 7.5 (1.6) points at baseline to 2.4 (2.4) points at the 24-week follow-up, with a difference, mean (SD) and [95% CI] of −5.2 (3.8) [−7.1 to −4.0], *p* < 0.001; VHI from a mean (SD) of 10.6 (3.3) points at baseline to 14.6 (0.5) points at the 24-week follow-up, with a difference, mean (SD) and [95% CI] of 4.2 (0.5) [3.1 to 5.2], *p* < 0.001; VuHI from a mean (SD) of 9.7 (3.1) points at baseline to 2.1 (1.8) points at the 24-week follow-up, with a difference, mean (SD) and [95% CI] of −7.6 (3.5) [−8.6 to −3.7], *p* < 0.001;vaginal pH from a mean (SD) of 6.5 (0.8) to 5.0 (2.1), with a difference, mean (SD) and [95% CI] of −1.3 (0.8) [−2.3 to −0.77], *p* < 0.001; VMI from a mean (SD) of 5.7 (9.2) to 18.4 (17.5), with a difference, mean (SD) and [95% CI] of 12.5 (8.3) [8.2 to 17.4], *p* < 0.001.

Unlike what was observed at 10 weeks of follow-up, at 24 weeks a statistically significant improvement was observed in EORTC QLQ-BR23 from a mean (SD) of 39.1 (8.4) to 43.3 (3.4), with a difference, mean (SD) and [95% CI] of 4.1 (4.2) [0.6 to 7.9], *p* < 0.001. Similarly, a significant improvement in FSFI was observed from 18.8 (5.1) to 23.7 (6.2) with a difference, mean (SD) and [95% CI] of 5.2 (2.2) [4.3 to 9.1], *p* < 0.001, and in the lubrication with a mean (SD) of 2.7 (1.4) to 4.3 (1.7) with a difference, mean (SD) and [95% CI] of 1.5 (1.1) [0.9 to 2.3], *p* < 0.001.

Complications related to the use of vaginal SSVL therapy were also recorded, and there were no major complications. Vaginal bleeding during the application of the laser decreased significantly from 70.3% in the first session compared to 3.7% in the third and 0% in the last session. Vaginal pain (0–10) during laser application (mean, SD) decreased significantly from 4.1 (2.4) to 2.2. (2.3), *p* < 0.001; similarly to the vulvar pain (0–10) with a media from 3.8 (2.1) in the first session vs. 1.8 (0.9), *p* < 0.001 in the last one. At 6-month follow-up satisfaction with treatment was very high (4.7 out of 5), with 95.7% of patients being satisfied, more than satisfied or very satisfied (Table 3).

## 4. Discussion

Laser and other energy-based devices have been marketed for the treatment of VVA, but the safety and efficacy of these devices remain uncertain [27]. In different systematic reviews and meta-analyses, CO_2_ and Er:YAG lasers have been shown to improve GSM, dyspareunia and sexual function in healthy menopausal women but the quality of the body of evidence is “very low” to support its use as one of the standards in the treatment of postmenopausal VVA [27,28,29,30].

At present, several follow-up, non-RCT studies(15 with a CO2 laser and 4 with an Er:YAG laser) in 710 and 126BCSs, respectively, although with heterogeneous clinical characteristics and different treatment protocols and study outcomes, have concluded that laser therapy improves VVA symptoms, with high satisfaction with laser procedures and a high rate of tolerability and reported no side effects in short-term follow-up; however, there is a lack of data regarding safety and BC relapse [16,31,32,33].

Similar to these studies but with a SSVL, we have also found a significant improvement in VVA symptoms (dyspareunia, Vaginal Health Index, Vulvar Health Index and FSFI). In addition, we have found an improvement in quality of life without significant side effects at short-term follow-up (6 months), with high satisfaction with the SSVL procedure among BCSs.

In the only RCT comparing vaginal laser vs. hyaluronic acid suppositories in 43 BCSs, at 3 months, the score on the VHI, urogenital atrophy, quality of life and sexual health had improved significantly in both groups (*p* = 0.001) [34]. Very recently (2023), the only RCT comparing CO2 laser therapy vs. sham laser treatment in 72 BCSs with GSM receiving AIs concluded that vaginal laser treatment was found to be safe after 6 months, but no statistically significant differences in efficacy were observed between both groups [35]. There are currently no studies comparing the CO_2_ and Erb:Yag lasers to establish if one is better than the other, though there appears to be equivalence. To date, no RCT comparing topical estrogen cream with a vaginal laser has been undertaken in BCSs, but in healthy menopausal women, such studies have shown equivalent efficacy.

The main contribution of this study is the use of a new type of non-ablative Solid-State Vaginal Laser (SSVL).The SSVL is based on a non-ablative procedure likened to the radiofrequency effect. Its handpiece is characterized by a larger spot that does not focus the beam on the surface but deeply penetrates vaginal tissues, thus reaching layers in which collagen fibers are more represented with greater effectiveness. It also avoids damaging the mucosal surface, in opposition to what happens using ablative and fractional ablative systems [36]. In addition, it uses a more efficient light source that dissipates less heat and thus requires a less powerful cooling system, making it a more sustainable alternative [37].

Only three studies with the SSVL have been published, but none in BCSs [38,39,40]. Dodero et al. assess the effect of this therapy in postmenopausal women who had failed to respond to estrogen therapy or had refused it, focusing on histological changes pre and post-treatment. In their first study [38], with eighty participants (mean age 57.2 ± 5.4 years), the severity and the presence of GSM symptoms decreased significantly, while the sexual function (as assessed by the FSFI) improved significantly. At 8-week follow-up, 70% of patients no longer complained of any symptoms, and only 5% reported having little benefit. The number of patients that at the beginning of the study had a FSFI score ≤ 26.55 (corresponding to sexual dysfunction) dropped dramatically from 92% to 44% after the second laser treatment and only 10% at the end of the study, so the SSVL was effective in 90% of the treated cases. The VHI score increased significantly after the completion of the study protocol, demonstrating a restoration of vaginal epithelial tropism in 95% of treated women. After the SSVL treatment, almost all patients (91%) affected by urge/stress urinary incontinence obtained a complete remission of symptoms. An interesting observation to emerge from these studies is that for treatments to be effective, their use must be recommended in the years around menopause. Similar to these authors, we meet a statistically significant improvement in FSFI score (18.5 (7.1) at baseline vs. 21.05 (4.2) at the 10-week follow-up vs. 23.7 (6.2) at the 24-week follow-up; *p* < 0.001). In addition, our study showed a statistically significant improvement in the VHI (10.4 (3.1) vs. 17.1 (3.9) vs. 14.6 (3.6), but we can observe how after 6 months of treatment the VHI worsens slightly compared to 10 weeks of follow-up, which could mean the need for a new treatment session in the short term. We have not analyzed urge/stress incontinence.

In 2019, some authors [39] demonstrated an improvement in histological features after SSVL by comparing tissue samples collected before and after treatment (amelioration was observed in order of thickening of the derma surface, phenomenon of glycogenic acanthosis, increased number and highness of papillae in chorion and in terms of reduction in the inflammatory tissue infiltrate) and at the same time an amelioration was observed on VHI score, VVA symptoms and sexual female function. These results were progressive with treatment going by and reached a peak after the fourth laser session and were time lasting, being unvaried 4 weeks after the end. A very important key point is the demonstration of inflammatory tissue reduction. So, a decrease in the lymphocytic and, in general in all inflammatory infiltrates of derma and chorion, can be observed, and these infiltrates are almost absent after the last laser session, meaning a restitution ad integrum not only from a histological but also from an inflammation point of view [16]. This is probably one of the main points acting in the GSM symptom reduction. The SSVL equipment used in this study was the same that we have used in our study, and it is based on a specific protocol, called Ladylift^®^, performed with a laser with a wavelength of 1470 nm (Eufoton–Trieste Italy) transmitted with a specific probe and parameters customized according to the patient. However, these multicenter observational studies have several limitations: lack of long-term follow-up, lack of randomization with sham treatment and/or standard treatment, and absence of a comparator (placebo or other active treatment). Besides this, the histological results together with the improvement of GSM symptoms as indicated by VHI, FSFI and ICIQ-UI SF scores prove a real favorable effect of the SSVL on tissue remodeling and revitalization that ends in a ‘rejuvenation’ effect from both histological and symptomatic point of view. Stabile et al., in 2022, analyzing the GSM and the lower urinary tract-related symptoms, using the Ladylift^®^ non-ablative laser technology for the treatment of menopausal vestibulodynia and genitourinary syndrome, observed an improvement in the VHI and a reduction in the NRS pain scale up to one month after the end of treatment. However, the beneficial effect tended to gradually decrease over time, suggesting the need to perform more therapy sessions for a longer period of time [40]. Similar to this, we see a statistically significant improvement in the Vulvar Health Index (9.7 (2.8) at baseline to 2.1 (1.8) at the 24-week follow-up).

A special situation is the treatment of GSM in BCSs. The benefits of maintaining sexual activity to help improve local vascularization and decrease symptoms of GSM should also be recommended for BCSs. The Solid-State Vaginal Laser (SSVL) and recombinant platelet-derived epidermal growth factors (RGFs) are new alternatives that improve female sexual dysfunction resulting from dyspareunia and could be an alternative for BCSs [37].

In our study, we found a significant improvement in different subjective and objective VVA criteria in BCSs with a history of use or AIs at 4 weeks after the end of treatment and even greater at 6 months after it began. We believe that the subjective and objective variables to assess the GSM must be differentiated, and not many of the objective outcomes are used in the recent literature evaluating the GSM. There is the possibility that some therapies present only subjective improvement, giving place to a possible placebo effect that is not being evaluated [41]. Therefore, we have evaluated the vaginal pH measurement (used as a part of the VHI calculation), recommended since 2003 by the FDA to objectively assess GSM changes. Vaginal pH was the only objective GSM parameter associated with vaginal hypoestrogenism that has been measured before and after the CO2 laser in some studies in BCSs, in all studies with the erbium laser and in our present study. Similarly to us, the Vaginal Maturation Index (VMI), the other supporting finding of GSM diagnosis which quantifies the percentages of parabasal, intermediateand superficial cellsindirectly estimating the pattern of tissue hypoestrogenism was considered only in one study with the CO2 laser [42]. The VMI was significantly increased in the laser group even after having completed the laser therapy, indicating the long-term effect of laser regeneration. The pH value decreased in both groups, the effect of the laser being more pronounced [42]. It can be hypothesized that the decrease is due to an increase in superficial cells and the thickness of the epithelium and consequently glycogen production. Glycogen in the mucous layer serves as a substrate for vaginal colonization by Doderlein’s lactobacillus, which produces lactic acid, which in turn regulates the low vaginal pH [43]. Similarly, we meet a significant increase in the VMI and a decrease in pH value after the use of the SSVL in these patients. An objective non-invasive tool to assess the severity of GSM signs, such as 3D high-frequency vaginal ultrasound (US), which allows measurement of the anterior and posterior walls from the vagina separately [44], or changes in the vaginal microbiota or vaginal inflammatory factors according to some evidence [45,46], were not used in our study.

The main strength of the current study is that it is the first study of a new type of non-ablative Solid-State Vaginal Laser (SSVL) treatment on the vaginal epithelium, dyspareunia, sexuality and quality of life in BCSs with atrophy induced by adjuvant aromatase inhibitor therapy and provides preliminary evidence of safety. An additional strength includes a relatively long-term follow-up, 24 weeks, which is longer than the other ablative laser studies.

There are several limitations to this study: It is an observational study but a non-randomized control trial, without a sham or CO2/Er:YAG laser comparison arm. It has a small sample size, but this study was conducted during the COVID-19 pandemic, making it difficult to recruit and follow up participants. GSM treatment for these women may help restore their sexual relationships, but in isolation may not be enough to restore them to their pre-breast cancer sexual state. Another limitation was the lack of information on partner status, including relationship duration and quality, as well as information on frequency and type of sexual activity at each time point. Therefore, the findings of the study are a hypothesis and larger prospective studies are needed. A double-blind, randomized controlled trial, comparing SSVL with CO2/Er:YAG laser therapy or sham laser treatment is needed to further establish the short- and long-term safety and efficacy of SSVL laser treatment in women with breast cancer. Therefore, we are considering continuing the same study in a prospective randomized trial.

## 5. Conclusions

Breast cancer survivors with a history of aromatase inhibitor adjuvant therapy after a non-ablative Solid-State Vaginal Laser (SSVL) treatment reported sustained improvement in vaginal atrophy, vaginal pH, dyspareunia, sexual function and in quality of life with no serious adverse effects and with a high level of satisfaction twenty-four weeks after initial treatment in this non-randomized pilot study. This may be a useful, non-hormonal method of ameliorating this distressing symptom complex for BCSs. Laser therapy may be considered in BCSs who prefer a non-hormone approach, but they must be counseled regarding the lack of long-term safety and efficacy data on the procedure. Finally, further research to define the safety and efficacy of different types of vaginal lasers (including SSLV), as well as to develop new therapies is critical to the management of GSM on BCSs

## Figures and Tables

**Table 1:** Demographic characteristics.

Characteristic		Participants, No. (%) 27 (100%)
Age at enrollment, mean (SD), y		57.17 (3.69)
Age at menopause, mean (SD), y		46.88 (3.98)
Age at BC diagnosis, mean (SD), y		45.78 (3.62)
Time since BC diagnosis, mean (SD), y		11.39 (3.31)
Type of menopause	Natural	6 (22.22)
	Induced	21 (77.77)
Parity (have children)		22 (81.40)
Smokers		2 (7.40)
Education level	Elementary degree	6 (13.3)
	High school degree	16 (59.25)
	College degree	5 (18.51)
Household income (EUR/month)	<600	0 (0)
	601–1200	4 (14.81)
	1200–3600	19 (70.38)
	>3600	4 (14.81)
Breast cancer stage	I	13 (48.14)
	II	14 (51.86)
Surgery	Conservative surgery	17 (62.96)
	Mastectomy	10 (37.03)
Breast reconstruction	Yes	4 (4.81)
	No	6 (22.22)
History of adjuvant therapy	Hormone therapy (IAs)	27 (100)
	Radiotherapy	19 (70.37)
	Chemotherapy	22 (82.48)

**Table 2:** Efficacy outcomes before and after laser treatment: 10-week and 6-month follow-up.

Outcome	Baseline (n = 27)	10-Week Follow-Up (n = 27)			24-Week Follow-Up (n = 24)		
	Mean (SD)		Difference, Mean (SD) [95% CI]	*p* Value ^a^		Difference, Mean (SD) [95% CI]	*p* Value ^a^
**Subjective outcomes**							
Dyspareunia (VAS) ^b^	7.5 (1.6)	3.4 (2.4)	−4.1 (2.2) [−5.6 to −3.4]	<0.001	2.4 (2.4)	−5.2 (3.8) [−7.1 to −4.0]	<0.001
Vaginal Health Index (VHI) ^c^	10.6 (3.3)	17.1 (3.9)	6.5 (3.6) [5.2 to 8.3]	<0.001	14.6 (3.6)	4.2 (0.5) [3.1 to 5.2]	<0.001
Vulvar Health Index (VuHI) ^d^	9.7 (3.1)	3.9 (3.2)	−5.8(3.6) [−7.6 to −4.5]	<0.001	2.1(1.8)	−7.6 (3.5) [−8.6 to −3.7]	<0.001
EORTC QLQ-BR23 ^e^	39.1 (8.4)	37.8 (6.4)	−1.3 (4.5) [−7.7 to −0.8]	0.29	43.3 (3.4)	4.1 (4.2) [0.6 to 7.9]	<0.001
FSFI ^f^ Lubrication ^g^	18.8 (5.1)	21.05 (4.2)	2.2 (0.7) [1.9 to 4.3]	0.13	23.7 (6.2)	5.2 (2.2) [4.3 to 9.1]	<0.001
	2.7 (1.4)	3.1 (1.5)	0.4 (1.2) [0.5 to 1.9]	0.19	4.3 (1.7)	1.5 (1.1) [0.9 to 2.3]	<0.001
**Objective outcomes**							
Vaginal pH	6.5 (0.8)	6.3 (1.0)	−0.2 (0.1) [−0.9 to −0.4]	<0.001	5.0 (2.1)	−1.3 (0.8) [−2.3 to −0.7]	<0.001
Vaginal Maturation Index (VMI) ^h^	5.7 (9.2)	17.2 (16.7)	11.5 (3.23) [7.4 to 15.6]	<0.001	18.4 (17.5)	12.5 (8.3) [8.2 to 17.4]	<0.001

^a^ *p* values are the mean differences in the variable values before and after treatment, assessed with the paired-samples *t* test. ^b^ Range, 0 to 10 points; higher scores indicating worse pain during sexual intercourse. ^c^ Range, 5 to 21; scores of 15 or lower indicate vulvovaginal atrophy. ^d^ Range, 0 to 24, with higher scores corresponding with greater vulvar atrophy. ^e^ Range, 0 to 100; higher scores indicate better quality of life. ^f^ Range, 0 to 36 points; lower scores indicate worse sexual dysfunction. ^g^ Range, 0 to 6; higher scores indicate better lubrication. (It is an item of FSFI). ^h^ Range, 5 to 25; higher scores indicate better vaginal trophism.

**Table 3:** Safety, tolerance and satisfaction with laser treatment.

Measure	T1 ^a^ n = 27	T2 ^b^ n = 27	T3 ^c^ n = 27	T4 ^d^ n = 27	T5 ^e^ n = 27	T6 ^f^ n = 24	*p* Value
Vaginal burns (%)	0 (0)	NA	NA	NA	0 (0)	0 (0)	>0.99 ^j^
Vulvar burns (%)	0 (0)	NA	NA	NA	0 (0)	0 (0)	>0.99 ^j^
Vaginitis (%)	0 (0)	NA	NA	NA	1 (3.7)	0 (0)	0.32 ^j^
Vaginal bleeding during laser application (%)	19 (70.3)	4(14.8)	1(3.7)	0 (0)	NA	NA	<0.001 ^j^
Vaginal pain during laser application ^g^ (mean, SD)	4.1 (2.4)	NA	NA	2.2 (2.3)	NA	NA	<0.001 ^j^
Vulvar pain during laser application ^g^ (mean, SD)	3.8 (2.1)	NA	NA	1.8 (0.9)	NA	NA	<0.001 ^j^
Satisfaction with the treatment ^h^ (mean, SD)	NA	NA	NA	NA	3.9 (1.4)	4.7 (1.5)	<0.001 ^j^
Satisfaction grade with the treatment ^i^ (n, %)	Not at all Satisfied				1 (3.7)	0 (0)	
	Partly Satisfied				1 (3.7)	1 (4.1)	
	Satisfied				4 (14.8)	5 (20.8)	
	More than Satisfied				9 (33.3)	7 (29.1)	
	Very Satisfied				12 (44.4)	11 (45.8)	0.19 ^k^

NA: not applicable. ^a^ T1: 1º time point (baseline and first treatment). ^b^ T2: 2º time point (second treatment). ^c^ T3: 3º time point (third treatment). ^d^ T4: 4º time point, at 6 weeks (fourth treatment). ^e^ T5: follow-up after 4 weeks from the last laser application. ^f^ T6: final follow-up at 24 weeks. ^g^ Range, 0 to 10 points; higher scores indicating worse vaginal or vulvar pain during laser application. ^h^ Level of satisfaction was assessed using a Likert scale, numbering 1 to 5 as an interval scale. ^i^ Grade of satisfaction was assessed using a Likert scale, with scores ranging from “Not at all Satisfied”, “Partly Satisfied”, “Satisfied”, “More than Satisfied”, “Very Satisfied”, numbering 1 to 5 as an interval scale with higher scores indicating more satisfaction. ^j^ Assessed with *t* test. ^k^ Assessed with Fisher exact test.

## Data Availability

The data presented in this study are available upon reasonable request to the corresponding author.
